# Wisdom in Older Adults Aging with a Mobility Disability

**DOI:** 10.1093/geroni/igaf122.3084

**Published:** 2025-12-31

**Authors:** Hye Soo Lee, Olivia Rojas, Wendy Rogers

**Affiliations:** University of Illinois--Urbana-Champaign, Champaign, Illinois, United States; University of Illinois Urbana-Champaign, Champaign, Illinois, United States; University of Illinois Urbana-Champaign, Champaign, Illinois, United States

## Abstract

Whereas wisdom is seen as a beneficial concept for navigating life, it lacks a clear definition in academia. Researchers have suggested investigating the contextual factors of wisdom, such as culture, to enhance our understanding of wisdom. We extended this approach by examining a population that shares a specific life context. We used interview data from the Aging Concerns, Challenges, and Everyday Solution Strategies (ACCESS) study. The sample was 60 adults aged 60-79 (M = 69.4, SD = 5.7) who have lived with a mobility disability for at least 10 years. There were 26 males and 34 females, 86.7% of whom identified as white. We conducted a thematic analysis on the advice participants would offer to a similar other. Our coding scheme included a priori codes based on existing wisdom literature (i.e., self-regulation; cognitive and meta-cognitive realm; social realm; and spirituality and religiosity) as well as additional codes we identified from the data (i.e., physical health; perseverance; attitudes about receiving help; and thinking about the act of giving advice). We found the theme dependence/independence, reflecting participants’ discussion of maintaining independence and accepting help. Most of the wisdom mentioned helped participants to be on their own; however, some participants discussed the wisdom of allowing oneself to depend. Wise people are often characterized as advisors, supporters, and givers. This study allowed us to capture the wisdom of dependence, which may benefit all individuals facing declines in functioning. We also discuss the implications of finding wisdom codes that are not widely recognized in the extant wisdom literature.

